# Evidence that COMT genotype and proline interact on negative-symptom outcomes in schizophrenia and bipolar disorder

**DOI:** 10.1038/tp.2016.157

**Published:** 2016-09-13

**Authors:** C L Clelland, V Drouet, K C Rilett, J A Smeed, R H Nadrich, A Rajparia, L L Read, J D Clelland

**Affiliations:** 1 Department of Pathology and Cell Biology, Columbia University Medical Center, New York, NY, USA; 2 Taub Institute for Research on Alzheimer's Disease and the Aging Brain, Columbia University Medical Center, New York, NY, USA; 3 Bellevue Hospital Center, New York, NY, USA; 4 Department of Psychiatry, New York University School of Medicine, New York, NY, USA; 5 Movement Disorders and Molecular Psychiatry, The Nathan S. Kline Institute for Psychiatric Research, Orangeburg, NY, USA

## Abstract

Elevated peripheral proline is associated with psychiatric disorders, and there is evidence that proline is a neuromodulator. The proline dehydrogenase (*PRODH*) gene, which encodes the enzyme that catalyzes proline catabolism, maps to human chromosome 22q11.2, a region conferring risk of schizophrenia. In the *Prodh*-null mouse, an interaction between elevated peripheral proline and another 22q11.2 gene, catechol-*O*-methyltransferase (*COMT*), on neurotransmission and behavior has been reported. We explored the relationship between fasting plasma proline levels and *COMT* Val^158^Met genotype on symptoms (positive, negative and total) in schizophrenia patients. In an exploratory study we also examined symptom change in patients with bipolar disorder. There was a significant interaction between peripheral proline and *COMT* on negative symptoms in schizophrenia (*P*<0.0001, *n*=95). In *COMT* Val/Val patients, high proline was associated with low Scale for the Assessment of Negative Symptom (SANS) scores. In contrast, high proline was associated with high SANS scores in patients carrying a Met allele. The relationship between proline and COMT also appears to modify negative symptoms across psychiatric illness. In bipolar disorder, a significant interaction was also observed on negative-symptom change (*P*=0.007, *n*=43). Negative symptoms are intractable and largely unaddressed by current medications. These data indicate a significant interaction between peripheral proline and *COMT* genotype, influencing negative symptoms in schizophrenia and bipolar disorder. That high proline has converse effects on symptoms by *COMT* genotype, may have implications for therapeutic decisions.

## Introduction

Schizophrenia symptoms are typically divided into positive, negative and cognitive clusters, along with mood symptoms.^1^ Positive symptoms, including hallucinations and delusions, show the greatest response to treatment, whereas cognitive symptoms, such as conceptual disorganization, and negative symptoms including avolition, blunted affect and social withdrawal, are largely unaddressed by current medications. Indeed, negative symptoms are among the most persistent and debilitating in schizophrenia, and contribute significantly to the huge personal and economic costs of severe psychiatric illness.^2^

Proline is a precursor of the neurotransmitter glutamate and may function as a central nervous system (CNS) neuromodulator (reviewed in Phang *et al.*^3^). Peripheral hyperprolinemia, which reflects CNS proline elevation,^4, 5, 6, 7, 8, 9^ has been associated with psychiatric disorders including schizophrenia.^10, 11, 12^ The proline dehydrogenase gene (*PRODH*) encodes proline oxidase (POX), the enzyme that catalyzes the first step in proline catabolism. The direct consequences of elevated proline for neurotransmission have been best demonstrated by work on the hyperprolinemic *Prodh*-null model.^7, 8^ In the presence of POX deficiency and elevated proline (peripheral and CNS), the mouse exhibits altered glutamate and dopamine signaling, including an enhancement of glutamatergic synaptic transmission, prefrontal dopamine transmission, and functional hyperdopaminergic responses.^8^ Genetic association studies have also implicated *PRODH*, not only in schizophrenia,^13^ but also in the etiology of endophenotypes associated with schizophrenia.^14^

*PRODH* maps to chromosome 22q11.2, a region associated with the highest known genetic risk for schizophrenia, aside from that shared by monozygotic twins. In addition, this location is also associated with the hemizygous microdeletion found in 22q11.2 deletion syndrome (22q11DS), and there is an increased risk of schizophrenia as well as other psychotic, mood-, obsessive compulsive- and autism spectrum disorders in 22q11DS patients.^15, 16, 17, 18, 19^ Approximately 37–50% of 22q11DS patients have significant elevation of fasting plasma proline.^20^

The catechol-*O*-methyltransferase gene (*COMT*) encodes the eponymous enzyme that methylates and inactivates catecholamines including dopamine, and also maps to 22q11.2, distal to *PRODH*. The *COMT* Val^158^Met functional polymorphism (substitution of valine (Val) to methionine (Met) at residue 158), has been extensively studied with regards to dopamine neurotransmission, because Val/Val homozygotes have prefrontal cortical (PFC) enzyme activity ~40% higher than Met/Met homozygotes and are considered to have concomitant lower PFC dopamine levels.^21, 22^ It has thus been suggested that the Val^158^Met polymorphism modulates cognitive functioning (reviewed in Bilder *et al.*^23^). Although *COMT* has been associated with psychotic and mood disorders including schizophrenia and bipolar disorder,^24, 25^ results have been inconsistent.^26^

A CNS functional interaction between *COMT* and *PRODH* has been proposed by Paterlini *et al.*, who suggested that significant cortical *Comt* upregulation in the *Prodh*-null mouse represents a compensatory response to increased PFC dopamine transmission, arising as a consequence of *PRODH* deficiency enhancing glutamatergic synaptic transmission.^8^ In addition, psychosis with positive symptoms,^20^ decreased smooth pursuit eye movement,^27^ and deficits in visual processing,^28^ have been associated with high levels of plasma proline in 22q11DS patients carrying the low-activity Met allele. Given these reports, and our finding of significantly elevated fasting peripheral proline in schizophrenia patients versus healthy controls,^11^ we hypothesized that *COMT* may interact with proline level, modifying symptom domains in patients with schizophrenia. We therefore tested for effect modification between the Val^158^Met *COMT* genotype and fasting peripheral proline on both positive and negative symptoms of schizophrenia. To support our primary finding of an interaction on negative symptoms, and because negative symptoms are present across psychiatric disorders,^29, 30^ in an exploratory study, we also assessed the relationship between proline and *COMT* on negative symptoms in bipolar disorder. Valproate (VPA) treatment is widely used in both disorders and increases peripheral proline levels,^31^ and so we further tested for a differential effect of VPA treatment, based upon *COMT* genotype.

## Materials and Methods

### Subjects

Schizophrenia and bipolar disorder patients, aged 18–65 years, were recruited from Bellevue Hospital Center (BHC), a primary care facility, servicing relatively short-stay inpatients with acute psychiatric needs. The diagnosis of all patients was confirmed using the Structured Clinical Interview for DSM IV Disorders. After description of the study to subjects, written informed consent was obtained in accordance with institutional review board regulations.

Demographics and group descriptive data for the schizophrenia sample are shown in Table 1. Although recruitment was not targeted by *COMT*, patients were well-matched across groups. For schizophrenia patients, recruitment was cross-sectional and independent of their duration of hospitalization. Psychiatric symptoms were measured using the Schedule for Assessment of Negative Symptoms (SANS), the Schedule for Assessment of Positive Symptoms (SAPS) and the Brief Psychiatric Rating Scale (BPRS). Fasting proline levels of a subset of the schizophrenia patients, those who did not receive treatment with VPA, were reported previously.^11^

Bipolar patients were recruited upon presentation at the BHC Comprehensive Psychiatric Emergency Program. Psychiatric symptoms in bipolar disorder patients were measured at an admission visit (visit 1), using the BPRS. At a follow-up inpatient ward visit (visit 2), fasting bloods were collected plus a repeat BPRS assessment performed. As for the schizophrenia sample, recruitment was not targeted by genotype, but subjects were well matched on demographic characteristics (Table 2) and medication use across genotype groups (Supplementary Table 1). In addition, as shown in Supplementary Figure 1, we tested for an association of elevated proline in bipolar disorder patients.

### Determination of fasting plasma proline

Fasting morning blood draws were performed and proline measured in μmol l^−1^ as reported.^11^

### Genotyping

DNA was extracted from blood using the Puregene Blood Core Kit (Qiagen, Valencia, CA, USA) and the *COMT* fragment containing the Val^158^Met polymorphism amplified using the 5′–3′ primers: 5′-ACTGTGGCTACTCAGCTGTG-3′ and 5′-CCTTTTTCCAGGTCTGACAA-3′. A step-down PCR was employed with an initial denaturation of 94 °C:15 min, then 12 cycles of 94 °C:30 s, 58 °C:45 s and 72 °C:30 s, followed by 31 cycles of 94 °C:30 s, 50 °C:45 s and 72 °C:30 s, with a final 72 °C:7 min extension. NlaIII recognizes and cleaves the amplicon into Val (114 bp) or Met (96 bp) fragments, visualized following electrophoreses. To confirm genotyping accuracy, 40% of samples were repeat assayed.

### Statistical analysis

Demographic and clinical characteristics were compared across genotypes, using ANOVA, Kruskal–Wallis and Mann–Whitney tests, *χ*^2^ or Fisher exact tests as appropriate. Genotype distributions were tested for Hardy–Weinberg equilibrium using an exact test.

Multivariate analysis of covariance was employed to test the hypothesis of an interaction effect between *COMT* genotype and the continuous predictor variable fasting plasma proline, on schizophrenia symptoms (total SANS, SAPS and BPRS scores). Estimates of the interaction coefficients were obtained from the multivariate regression model, and tested for significance across the three dependent variables, with Bonferroni correction for *post hoc* comparisons. Homogeneity of variance and covariance matrices assumptions were confirmed (*P*>0.05) using Levine's and Box-M tests, respectively. Specific significant interaction effects that remained were assessed further via multivariable regression. To evaluate and then control for potential confounds between demographic and/or clinical characteristics with the dependent variable(s), covariates were entered into a bivariate linear regression and terms found to have *P*-values <0.10 carried forward to a multivariable model. Gender was a covariate in all models, to adjust for previously reported proline gender differences.^10, 11, 31^ Multivariable model fit and selection was determined via Wald tests, testing the null hypothesis that non-significant (*P*>0.05) covariate parameters were simultaneously equal to zero in full and subsequent reduced model. On the basis of the result from the schizophrenia study, in an exploratory analysis the primary outcome for bipolar patients was the BPRS-negative-symptom subscale,^32^ and percent reduction in negative symptoms calculated (for bipolar disorder patients, the range of BPRS scores at the blood draw visit (range 5–8) did not allow for cross-sectional analysis). When outliers in the data or leverage points were identified, a robust regression procedure using an MM estimator to minimize data point effects (SASv9.3) was employed. All statistical tests were two-tailed. Means and s.d. are reported. Statistical analysis was performed in SASv9.3 and Stata ICv12, with graphs plotted in GGplot2v1.0.1 in Rv3.1.2.

## Results

### *COMT* genotype modifies the relationship between proline and negative symptoms of schizophrenia

The schizophrenia sample (*n*=95) was well-matched across genotype groups, and similar in size to previously published studies in 22q11DS reporting a significant *COMT* × proline interaction.^27, 28^ There were no differences in BPRS total or negative symptoms (SANS total score, Table 1). As previously reported^33^ Met/Met patients had significantly lower SAPS scores than Val/Met (Mann–Whitney *z*=2.52, adjusted *P*=0.035) or Val/Val patients (*z*=2.92, adjusted *P*=0.001). We achieved 100% accuracy from confirmatory regenotyping and a sample of 90 control subjects were in Hardy–Weinberg equilibrium for *COMT* Val^158^Met (*P*>0.05, data not shown). *COMT* distributions of the schizophrenia patients deviated from Hardy–Weinberg equilibrium (*χ*^2^=8.08, df=1, *P*<0.05), which has been previously reported for this polymorphism in schizophrenia.^34^

Testing the primary hypothesis of an interaction effect on schizophrenia symptoms; results of the multivariate analysis of covariance for symptom scores showed a significant genotype × proline interaction, Wilk's *λ*=0.69, F_(2,174)_=6.01, *P*<0.0001 (Supplementary Table 2). Follow-up Wald tests with Bonferroni correction identified significant interaction effects specific to total SANS scores F_(2,89)_=13.33, adjusted *P*<0.001 but no significant interaction effect on total SAPS F_(2,89)_=2.97, adjusted *P*=0.168, or BPRS scores F_(2,89)_=0.42, adjusted *P*=1.0, suggesting specificity of the relationship to negative symptoms.

**Figure 1:**
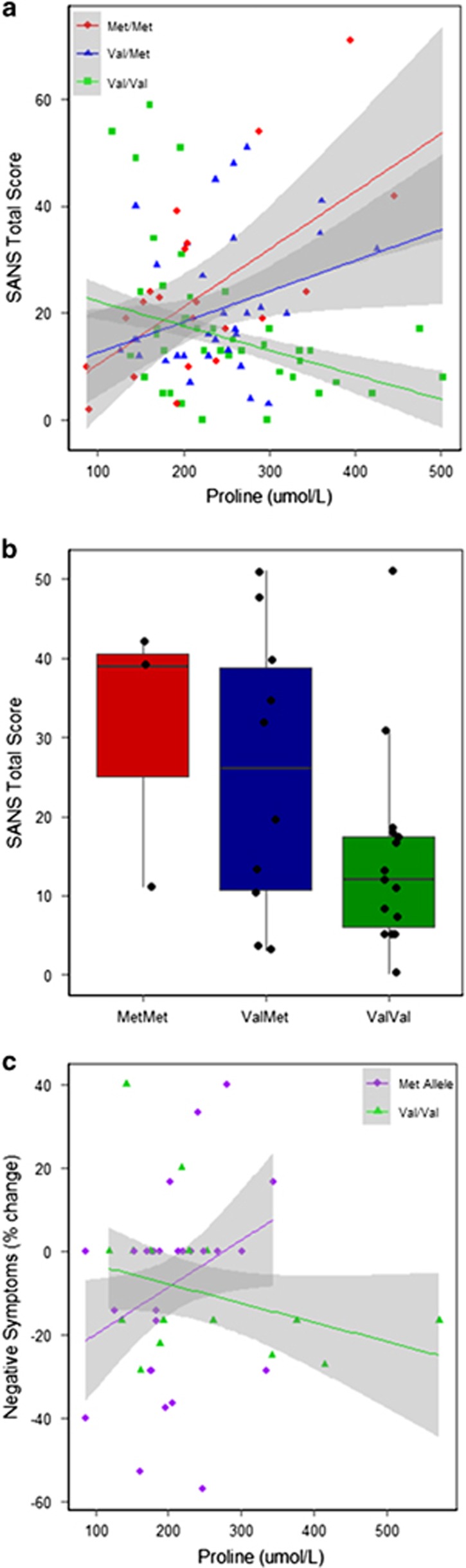
(**a**). The Interaction between proline and catechol-*O*-methyltransferase (*COMT*) genotype on negative symptoms in schizophrenia. There is a significant interaction between proline and *COMT* Val^158^Met genotype on total SANS score (Initial Model, Table 3, *P*<0.001). The graph shows this relationship plotted for patients with the Met/Met (*n*=21, red diamonds), Val/Met (*n*=32, blue triangles) and Val/Val (*n*=42, green squares) genotypes. Lines represent the predicted values from the regression model for each genotype, with 95% confidence intervals shown. In both Met/Met and Val/Met patients, high proline levels are associated with high SANS scores. Conversely, there is a significant negative relationship in Val/Val patients, with high proline associated with lower levels of negative symptoms. (**b**) Negative symptoms in schizophrenia patients treated with valproate (VPA), by *COMT* genotype. The boxplot illustrates that negative symptoms, as assessed by SANS score, are significantly lower in VPA-treated patients with the Val/Val genotype (mean=14.6±12.67. *n*=15, green), as compared with grouped Met/Met (mean=30.67±17.1, *n*=3, red) and Val/Met (mean= 25.6±17.93, *n*=10, blue) genotypes (F(_1,26_)=4.63, *P*= 0.0408). Subjects were included in this analysis if they had received 48 h or more of VPA treatment,^35^ within 48 h of the study visit, *n*=28 (three subjects were dropped as they had <48 h of VPA treatment). The actual minimum period of VPA treatment was 96 h. Black jittered points represent individual data. The horizontal line within each box represents the group median. The box indicates the IQR. The whiskers extend to the most extreme data point which is 1.5 × the IQR. (**c**) The interaction between proline and *COMT* genotype on percent change in negative symptoms in bipolar disorder. There is a significant interaction between proline and *COMT* Val^158^Met genotype on the percent reduction in negative symptoms (Initial Model Table 4, *P*=0.04). The graph shows the relationship plotted for patients with the Met allele (*n*=27, purple diamonds) and Val/Val genotype (*n*=16, green triangles). Lines represent the predicted values from the regression model. As proline rises Val/Val patients exhibit a greater percent reduction in their negative symptoms. Conversely, in Met allele carriers, increasing proline corresponds to lack of improvement, with unchanging or increasing negative symptoms. Negative symptoms were evaluated using the Brief Psychiatric Rating Scale subscale^32^ consisting of items: 3 (emotional withdrawal); 13 (motor retardation); 14 (uncooperativeness); 16 (blunted affect); and 18 (disorientation). Percent change in symptoms was calculated using the following formula: ((negative symptoms at visit 2−negative symptoms at visit 1)/negative symptoms at visit 1) × 100%. IQR, interquartile range; SANS, Scale for the Assessment of Negative Symptom.

To examine the significant multivariate analysis of covariance on total SANS scores, interaction effects were graphed in Figure 1a (Supplementary Figure 2). For schizophrenia patients with both the Met/Met and Val/Met genotypes, high proline was associated with high SANS scores, while conversely high proline in Val/Val patients was associated with lower-negative-symptom scores. Stratifying by *COMT* (Met allele carrier or Val/Val), for Met carriers every 100 μM increase in proline (~1 s.d. from the mean proline level), was associated with a SANS total score increase of over 8 points (*β* coefficient=0.084, *P*=0.001). Conversely, for Val/Val patients every 100 μM increase in proline decreased SANS total scores by nearly 7 points (*β*=−0.067, *P*=0.003). Thus, at proline levels only ~1 s.d. above the group means, Met carriers with a fasting plasma proline of 332 μM have a predicated SANS score of 30, whereas Val/Val patients with proline of 346 μM have a predicted score of only 10. The significant interaction remained following stratified analysis of ethnicity, which was found not to influence the interaction effect (Supplementary Table 3), stratification and then adjustment for duration of illness (determined as the period since the subjects' first hospitalization for psychiatric symptoms; Supplementary Table 4), and after removal of patients reporting alcohol abuse/dependence (*P*<0.005, *n*=72). Supporting our SANS finding, we also observed a significant interaction between *COMT* (Met carriers versus Val/Val patients) and high proline on the negative-symptom subscale of the BPRS (interaction *β*=−0.0266, *P*=0.002).

Possible confounds on the *COMT* × proline interaction on total SANS score were assessed. Covariate analysis showed no relationship between SANS score and medication type, neuroleptic dose (summarized as daily chlorpromazine equivalents), duration of illness or the number of days in hospital prior to blood draw and symptom assessment (Supplementary Table 5). However, there was a relationship between SANS and ethnicity and alcohol use (*P*<0.1) and along with gender these variables were taken forward to a multivariable model (Table 3). Model fit was determined with the final model retaining genotype (ordinal due to the graphed relationship observed in Figure 1a), proline, alcohol use and the highly significant *COMT*-proline interaction (*P*<0.0001).

### Valproate-treated *COMT* Val/Val schizophrenia patients have significantly lower-negative symptoms than Met allele carriers

An effect of VPA on plasma proline has been reported^31^ and VPA-treated schizophrenia patients in our study had significantly higher proline (mean: 299.29±94.76, *n*=28) than those who did not receive VPA (mean: 215.84±63, *n*=64*; z*=−3.97, *P*=0.0001). Considering our finding of an interaction between *COMT* and proline on negative symptoms, we next hypothesized that VPA-treated Val/Val patients would respond differently to the concomitant higher levels of proline, with respect to their negative symptoms, as compared with Met carriers. As shown in Figure 1b, VPA-treated Val/Val schizophrenia patients had significantly lower SANS total scores, averaging 12 points lower than Val/Met and Met/Met patients (*β*=−12.17, *P*=0.041, *n*=28). This result remained significant after adjusting for the dose of VPA administered in the 48 h prior to the blood draw (*P*=0.043).

### *COMT* genotype modifies the relationship between proline and negative-symptom change in bipolar disorder

As an exploratory analysis, we employed a second patient sample: 43 subjects with bipolar disorder who had completed a BPRS assessment upon admission to the psychiatric ER (visit 1) plus a second BPRS assessment and fasting blood draw during their follow-up visit (mean duration between assessments=9.5±4.6 days). Thus, for this sample we were able to explore the relationship between *COMT* and proline on the change in symptoms, calculating the percent reduction in negative symptoms from admission to follow-up.

The distribution of *COMT* genotypes was in Hardy–Weinberg equilibrium (*χ*^2^=0.387, df=1, *P*>0.05). Due to the finding in schizophrenia that Met allele carriers have a similar response to high proline, and because of the smaller bipolar sample size, Met/Met and Val/Met bipolar patients were grouped together for further analysis.

We observed a significant interaction between *COMT* and fasting peripheral proline on the percent change in negative symptoms (interaction coefficient=−0.0017, *P*=0.04, Table 4). As shown in Figure 1c, high proline was associated with a greater reduction of negative symptoms for Val/Val bipolar patients, but conversely, Met carrier patients with high proline levels had in general either no change or a positive change in symptoms, suggesting a worsening of symptoms over time. Again, possible confounds were assessed (Supplementary Table 6). Regarding medication, although there was no relationship between change in negative symptoms and mood stabilizer use or neuroleptic dose, covariate analysis indicated that neuroleptic type and benzodiazepine use, plus the duration between visits, were predictors of the change in negative symptoms, as were the demographic characteristics of ethnicity and gender (*P*<0.1, Supplementary Table 6). These covariates were taken forward to multivariable models (Table 4). Sequential Wald Tests were performed, determining goodness-of-fit, with the proline–*COMT* interaction remaining significant after adjustment for gender in the final model (interaction *β*=−0.0021, *P*=0.0007).

As we found with the schizophrenia sample, bipolar VPA-treated patients had significantly higher fasting plasma proline than those who did not receive VPA (Supplementary Figure 3). However, although Val/Val-treated patients had a greater overall reduction in negative symptoms compared with Met carriers, this result did not reach significance likely due to the small sample.

## Discussion

The data presented in this study demonstrate that fasting peripheral proline and *COMT* Val^158^Met genotype, predict negative-symptom severity in schizophrenia. Specifically, we present evidence that for inpatients with the Val/Val genotype (encoding the high-activity COMT enzyme), high proline was associated with lower levels of negative symptoms: as proline rose across the Val/Val patients, negative symptoms decreased. Conversely, Met allele carriers displayed the opposite relationship, exhibiting significantly more negative symptoms as proline levels rose. Over the range of fasting proline in our schizophrenia sample (87–502 μm), this represents a significant and clinically relevant difference in negative symptoms between *COMT* genotype groups.

VPA upregulates circulating proline and, in our study VPA-treated schizophrenia Val/Val patients had significantly less-negative symptoms than VPA-treated Met allele patients, likely due to the impact of VPA on proline level. Our data have implications for treatment decisions, because proline-modulating medications, such as VPA which is commonly used to treat schizophrenia patients, may have beneficial, and conversely detrimental effects on negative symptoms, based upon the individual patient's Val^158^Met genotype.

In a second sample; inpatients with bipolar disorder, we explored the interaction between *COMT* and proline on negative-symptom change (using the BPRS-negative-symptom subscale). Supporting our earlier schizophrenia finding, we observed a significant interaction between proline and *COMT*: high proline was associated with improvement of negative symptoms in homozygous Val/Val bipolar disorder patients, whereas high proline in Met allele carriers was associated with less improvement or an increase in negative-symptom severity. This finding was not confounded by medication use, the duration of time between assessments, or demographic characteristics of the bipolar sample. Interestingly, the bipolar patients did not have proline levels significantly higher than controls, suggesting that in contrast with schizophrenia,^11^ proline may impact negative symptoms and their severity, but not bipolar disorder risk.

To our knowledge, this is the first study to document that proline and *COMT* interact to predict negative-symptom outcomes in psychiatric disorders. Our findings of a detrimental effect of high proline in combination with the *COMT* Met allele, on schizophrenia and bipolar disorder negative symptoms, is in part supported by studies of 22q11DS patients, who have an increased risk of psychosis (albeit exhibiting positive symptoms^20^) plus a neurophysiological visual sensory deficit,^28^ when carrying the Met allele in the presence of high proline. Moreover, Hidding *et al.*^36^ recently reported the negative effect of high proline and hemizygous *COMT*^Met^ on autism spectrum symptoms in children and adolescents with 22q11DS, which is particularly relevant given the notable symptom similarities between autism and schizophrenia spectrum disorders.^37, 38^

Our finding that high proline is protective in Val/Val patients with schizophrenia and bipolar disorder is also novel and significant. Intriguingly, Zarchi *et al.*,^39^ had reported the protective effect of a *PRODH* variant (the Tryptophan (Trp) allele of the Arg^*185*^Trp polymorphism) on a neurophysiological measure; mismatch negativity in *COMT*^Val^ 22q11DS patients. As the Trp allele exhibits decreased POX activity *in vitro,*^40^ Zarchi *et al.*, discussed either an opposite effect of this allele *in vivo*, or alternatively that the Arg^*185*^Trp polymorphism is in linkage disequilibrium with another functional SNP;^39^ in each circumstance likely resulting in increased POX activity and low peripheral proline. Our current study data suggest the opposite to that interpretation: That high proline is actually protective in hemizygous 22q11DS patients with the Val genotype, with regards to mismatch negativity.

Putative CNS roles of proline have been described both in terms of its potential as a neurotransmitter, as suggested by its uptake into and direct synthesis within synaptosomes and its release at the synapse after K+ induced depolarization,^3, 41, 42, 43^ as well as a neuromodulator of neurotransmitter systems, as suggested by the presence of high-affinity proline transporters in glutamatergic neurons,^3, 44, 45, 46^ and the enhancements of glutamatergic and prefrontal dopamine transmission in the presence of *Prodh* deficiency and elevated proline.^8^ Moreover, proline metabolism to Δ1-pyrroline-5-carboxylate (P5C) and then glutamate, produces ATP, reactive oxygen species (particularly superoxide radicals), and the conversion of NAD^+^ to NADH, initiating multiple downstream metabolic signaling pathways.^47, 48^ Although the mechanism by which proline elevation may impact neurotransmission requires further investigation, it is apparent from studies demonstrating alterations in the cell redox state and oxidative stress in conditions of high proline (reviewed in Wyse *et al.*^49^), the *Prodh*-null model,^7, 8^ as well as the human hyperprolinemias^3^ and pyrroline-5-carboxylate reductase deficiencies,^50, 51^ that elevated proline and/or abnormalities in proline metabolism and biosynthesis, can be detrimental to the CNS.

In schizophrenia and bipolar disorder, presence of the *COMT* Met allele may further accentuate proline toxicity. For example, in one putative model, enhanced dopamine-transmission in the PFC as a result of excess proline is exacerbated by low COMT activity and concomitant higher prefrontal dopamine availability, ultimately resulting in a frontal hyperdopaminergic state that mimics the *Prodh*-null mouse^8^ (and reviewed in Drew *et al.*^52^). A hyperdopaminergic model influencing negative symptoms is somewhat counterintuitive, given that negative symptoms are generally considered to arise from deficient mesocortical dopamine stimulation. However, *COMT* is involved in maintaining PFC cognitive stability^23, 53^ and in situations of high cortical dopamine concentrations and D_1_ receptor stimulation (likely present in Met/Met and to a lesser degree Val/Met psychiatric patients), enhanced cognitive stability of neuronal network activation has been theorized to result in a cognitive rigidity that may increase the likelihood of negative symptoms.^23^

Conversely, we found that proline elevation beneficially influences negative-symptom outcomes in Val/Val patients. In a *COMT* Val homozygous state, high enzymatic activity in the PFC would likely reduce prefrontal dopamine, limiting D_1_ receptor-mediated excitation.^23, 53^ Speculatively, proline elevation may increase prefrontal dopamine signaling, through interference with glutamatergic pathways, reducing vulnerability to a prefrontal hypodopaminergic state in Val/Val patients. Although *COMT* genotype may also interact with proline-induced altered cellular redox and increased oxidative stress, we consider that a parsimonious interpretation of currently available information regarding neuromodulatory effects of proline^3^ suggests that in the presence of elevated proline, negative symptoms are significantly impacted in conditions of both hyper- or hypodopaminergia, which is consistent with the PFC functioning model theorized by Mattay *et al.*, in which the optimal balance of dopamine signaling falls within a narrow range, following an inverted U-shaped curve.^54^

Interestingly, we found no relationship between *COMT* and proline on positive symptoms in schizophrenia. Positive symptoms are considered to arise from hyperactive subcortical mesolimbic projections, and our finding is consistent with the action of proline in murine cortical but not striatal dopamine potentiation.^8^ In addition, dopamine transporters are relatively sparse in the PFC,^55^ and the removal of dopamine there may be more impacted by COMT activity and the interaction with proline, as compared with subcortical regions.

Some study limitations exist: in the schizophrenia sample, proline was measured and symptoms assessed cross-sectionally. Thus, our findings may be confounded by enrollment differences across genotypes, and one particularly relevant variable to consider regarding this limitation is the duration of illness and how the structure of positive and negative symptoms may change during the course of the illness. In our study, although the duration of illness was higher in Met/Met patients, negative symptoms were not significantly different between genotypes, there was no significant main effect of *COMT* on negative symptoms, and the length of current hospitalization prior to symptom assessment had no relationship with negative symptoms. Furthermore, the significant proline × *COMT* interaction remained following analysis performed in a bisected sample of short and long illness durations. Taken together, we consider that the cross-sectional nature of the study did not significantly confound our findings. In addition, although the bipolar study allowed initial investigation of symptom change, the bipolar sample size was smaller and negative symptoms were assessed using a subscale of the BPRS. Further research would therefore benefit from a longitudinal approach, investigating the interaction between proline and *COMT* on the change in negative symptoms assessed via the SANS or Positive and Negative Symptom Scale, in a large sample of both schizophrenia and bipolar disorder patients.

Nonetheless, there are currently no medications approved for the treatment of negative symptoms in psychiatric illness, which are associated with poor functional outcomes and quality of life, are highly persistent, and are a great burden for caregivers.^2^ Our finding of a beneficial effect on negative symptoms of high proline in Val/Val inpatients suggests that personalization of treatments based upon a patient's *COMT* genotype, for the purpose of up- or downregulating proline level should be further investigated, as it may hold promise as a pharmacogenomics approach to intervene and target this symptom domain.

## Figures and Tables

**Table 1:** Demographics of schizophrenic sample, *n*=95

*Characteristics*	*Met/Met* n*=21*	*Val/Met* n*=32*	*Val/Val* n*=42*	P*-value*^a^
*Gender*, n *(% )*				0.288
Female	11 (23.4)	19 (40.4)	17 (36.2)	
Male	10 (20.8)	13 (27.1)	25 (52.1)	
				
*Ethnicity*, n (%)				0.096
African American	5 (13.5)	10 (27.0)	22 (59.5)	
Caucasian	10 (35.7)	10 (35.7)	8 (28.6)	
Hispanic	6 (20.0)	12 (40.0)	12 (40.0)	
				
Age (years), mean±s.d.	40.9±10.9	39.1±11.5	39.9±11.6	0.820
				
*History of alcoholism*, n (%)				0.426
Neither	17 (23.6)	27 (37.5)	28 (38.9)	
Abuse	1 (10.0)	2 (20.0)	7 (70.0)	
Dependence	3 (23.1)	3 (23.1)	7 (53.8)	
				
Duration of Illness (years),^b^ mean±s.d.	19.67±12.62	13.11±10.99	11.5±10.23	0.136
Hospital duration (days),^c^ mean±s.d.	19.1±17.1	21.9±23.4	20.0±19.6	0.998
Fasting plasma proline, μmol l^−1^	219.9±91.6	240.5±68.6	246.4±91.1	0.391
				
*Symptoms*				
BPRS^d^ total symptoms, mean±s.d.	32±8.5	33.6±7.1	33.6±8.4	0.500
SAPS^e^ total symptoms, mean±s.d.	10.3±8.3	15.8±9.6	18.2±10.1	0.006^a^
SANS^f^ total symptoms, mean±s.d.	24±16.8	21.8±13.1	17.5±13.9	0.127
				
*Neuroleptic medications*				
Neuroleptic type, *n* (%)				0.348
Typical only	5 (27.8)	3 (16.7)	10 (55.6)	
Atypical only	13 (22.4)	19 (32.8)	26 (44.8)	
Both	3 (16.7)	9 (50.0)	6 (33.3)	
Daily CPZE dose,^g^ mean±s.d.	490.6±234.0	571.1±418.1	526.8±281.0	0.981
				
*Mood-stabilizing medications*				
Mood stabilizer yes; *n* (%)	15 (26.3)	19 (33.3)	23 (40.4)	0.443
VPA treatment yes; *n* (%)	4 (12.9)	11 (35.5)	16 (51.6)	0.327
				
*Other medications*				
Benzodiazapines, yes: *n* (%)	4 (21.0)	8 (42.1)	7 (36.8)	0.641
Antidepressants, yes: *n* (%)	1 (9.1)	5 (45.4)	5 (45.4)	0.596

Abbreviation: COMT, catechol-*O*-methyltranserase; CPZE, chlorpromazine.

a Significant *P*-value when comparing characteristics across COMT genotypes.

b Determined as the period since the subjects' first hospitalization for psychiatric symptoms, *n*=60 for whom this characteristic could be obtained.

c Days in hospital prior to fasting blood draw.

d Brief Psychiatric Rating Scale.

e Schedule for Assessment of Positive Symptoms.

f Schedule for Assessment of Negative Symptoms.

g CPZE equivalent dose, *n*=94 (as one subject's NL had no CPZ equivalent).

**Table 2:** Demographics of bipolar disorder sample, *n*=43

*Characteristics*	*Met/Met* n*=5*	*Val/Met* n*=22*	*Val/Val* n*=16*	P*-value*^a^
*Gender*, n (%)				0.328
Female	1 (6.2)	11 (68.8)	4 (25.0)	
Male	4 (14.8)	11 (40.7)	12 (44.4)	
				
*Ethnicity*, n (%)				0.450
African American	0	4 (57.1)	3 (42.9)	
Asian	0	0	1 (100)	
Caucasian	3 (11.5)	13 (50.0)	10 (38.5)	
Hispanic	2 (22.2)	5 (55.6)	2 (22.2)	
				
Age (years), mean±s.d.	34±9.7	32.8±8.4	33.2±11.2	0.933
				
*History of Alcoholism, n (%)*				1.000
Abuse	1 (7.1)	8 (57.1)	5 (35.7)	
Dependence	1 (12.5)	4 (50.0)	3 (37.5)	
Neither	3 (14.3)	10 (47.6)	8 (38.1)	
				
Fasting proline,^b^ μmol l^−1^	213.6±72.7	205.5±63.2	245.8±123.4	0.669
				
Illness duration (years)^c^, mean±s.d.	5.5±6.8	4.9±6.0	9.3±8.7	0.304
				
Days between symptom assessments, mean±s.d.	10.2±6.2	9.4±3.7	9.5±5.1	0.382

a *P*-value when comparing Met allele carriers to Val/Val patients.

b Sampled at visit 2.

c Determined as the period since the subjects' first hospitalization for psychiatric symptoms, *n*=35 for whom this characteristic could be obtained.

**Table 3:** Results: the interaction between proline and *COMT* predicts negative symptoms in schizophrenia

	*β coefficient*	*s.e.*	*Test statistic*^a^	P*-value*	*Wald test*
Dependent Variable=Total SANS Score, *n*=95					

*Initial Model*^b^					
Proline	−0.0870	0.0317	7.55	0.0002*	
COMT (ordinal Val/Val, Val/Met, Met/Met)	−9.7339	4.0994	5.64	0.0176*	
Interaction (COMT × proline)	0.0560	0.0167	13.06	0.0003*	
					
*Full Model*^b^					
Proline	−0.1050	0.0333	9.94	0.0016*	
COMT (ordinal Val/Val, Val/Met, Met/Met)	−13.0179	4.2452	9.40	0.0022*	
Interaction (COMT × proline)	0.0744	0.0169	19.48	<0.0001*	
* *Alcohol use					
Alcohol abuse vs none	−4.2178	4.2706	0.98	0.3233	
Alcohol dependence vs none	−9.0807	3.5632	6.49	0.0108*	
** Gender**	**−1.0466**	**2.3795**	**0.19**	**0.6600**	
** Ethnicity**					
** African American vs Caucasian**	**3.0258**	**3.0258**	**1.14**	**0.2866**	
** African American vs Hispanic**	**4.6703**	**2.8214**	**2.74**	**0.0979**	***P*=0.517**^c^
					
*Final Model*^b^					
Proline	−0.0804	0.0321	6.28	0.0122*	
COMT (ordinal Val/Val, Val/Met, Met/Met)	−9.6576	4.0300	5.74	0.0166*	
Interaction (COMT × proline)	0.0651	0.0161	16.39	<0.0001*	
* Alcohol use*					
* Alcohol abuse vs none*	*−5.1234*	*3.9854*	*1.65*	*0.1986*	
* Alcohol dependence vs none*	*−9.7478*	*3.3526*	*8.45*	0.0036*	*P=0.020*^d^

Abbreviations: COMT, catechol-*O*-methyltransferase; SANS, Scale for the Assessment of Negative Symptom.

a χ^2^ (Schizophrenia models using Robust linear regression).

b Robust regression, MM Estimation Method.^56^

c Robust Wald test: canonical linear hypothesis that combined effect of covariates (Gender and Ethnicity, bold) is zero.

d Robust Wald test: covariate effect (Alcohol use, italics) is zero.

**Table 4:** Results: The interaction between proline and *COMT* predicts negative-symptom change in bipolar disorder

	*β coefficient*	*s.e.*	*Test statistic*^a^	P*-value*	*Wald test*
Dependent Variable=% Change in BPRS Negative Symptoms Scale, *n*=43					
					
*Initial Model*					
Proline	0.0011	0.0006	1.69	0.099	
COMT (Met allele vs Val/Val)	0.3834	0.1862	2.06	0.046*	
Interaction (COMT × proline)	–0.0017	0.0007	–2.42	0.040*	
					
*Full Model*					
Proline	0.0012	0.0006	2.09	0.044*	
COMT (Met Allele v Val/Val)	0.4281	0.1650	2.60	0.014*	
Interaction (COMT × proline)	–0.0017	0.0007	–2.42	0.022*	
Gender	0.1960	0.0656	2.99	0.005*	
** Ethnicity**					
** African American vs Caucasian**	**0.0186**	**0.0839**	**0.22**	**0.826**	
** African American vs Hispanic**	**–0.1528**	**0.1052**	**–1.45**	**0.156**	
** Duration (days) between assessments**	**0.0049**	**0.0070**	**0.69**	**0.492**	
** Neuroleptic type**					
** Atypical neuroleptic vs none**	**–0.0802**	**0.0818**	**–0.98**	**0.334**	
** Typical Neuroleptic vs none**	**–0.1401**	**0.2087**	**–0.67**	**0.507**	
** Both vs none**	**–0.1531**	**0.1184**	**–1.29**	**0.206**	
** Benzodiazepines**	**–0.0840**	**0.0714**	**–1.18**	**0.249**	***P*=0.056**^b^
					
*Final Model*					
Proline	0.0016	0.0006	2.55	0.015*	
COMT (Met Allele vs Val/Val)	0.5029	0.1766	2.85	0.007*	
Interaction (COMT × proline)	–0.0021	0.0007	–2.83	0.007*	
* Gender*	*0.1856*	*0.0656*	*2.83*	0.007*	*P=0.0074*^c^

Abbreviations: BPRS, Brief Psychiatric Rating Scale; COMT, catechol-*O*-methyltransferase.

a *t*(bipolar models using linear regression).

b Wald test: canonical linear hypothesis that combined effect of covariates (Ethnicity, Duration, Neuroleptic Type and use of Benzodiazepines, bold) is zero.

c Wald test: covariate effect (Gender, italics) is zero.
