# Supplementary material for: Evidence that COMT genotype and proline interact on negative-symptom outcomes in schizophrenia and bipolar disorder
Source: Transl Psychiatry. 2016 Sep 13;6(9):e891–. doi: 10.1038/tp.2016.157 (PMC5048199; doi:10.1038/tp.2016.157)
Supplement: Supplementary Information [file tp2016157x1.doc]

| **Supplementary Information.**  **Supplementary Table 1. Bipolar Disorder Sample: Assessment Scores and Treatment, by Visit (n=43).** | | | | | | | | | |
| --- | --- | --- | --- | --- | --- | --- | --- | --- | --- |
| **Characteristic** | **Admission (Visit 1)** | | | |  | **Follow-up (Visit 2)** | | | |
|  | **Met/Met** | **Val/Met** | **Val/Val** | **p-value^1^** |  | **MetMet** | **Val/Met** | **ValVal** | **p-value^1^** |
|  | *n=5* | *n=22* | *n=16* |  |  | *n=5* | *n=22* | *n=16* |  |
| **Brief Psychiatric Rating Scale** |  |  |  |  |  |  |  |  |  |
| Total Symptoms, mean ± SD | 42 ± 7.3 | 36.3 ± 5.9 | 36.4 ± 4.7 | 0.592 |  | 34.8 ± 8.8 | 25.9 ± 5.8 | 27.4 ± 5.2 | 0.772 |
| Negative Symptoms^2^, mean ± SD | 9.0 ± 5.1 | 6.3 ± 2.2 | 6.2 ± 1.7 | 0.872 |  | 6.0 ± 1.4 | 5.6 ± 0.9 | 5.6 ± 1.0 | 0.711 |
| Positive Symptoms^3^, mean ± SD | 24.6 ± 5.0 | 18.7 ± 6.2 | 18.2 ± 6.2 | 0.457 |  | 18.4 ± 5.3 | 12.4 ± 4.8 | 13.7 ± 4.2 | 0.553 |
|  |  |  |  |  |  |  |  |  |  |
| **Neuroleptic (NL) Medications** |  |  |  |  |  |  |  |  |  |
| NL Type, n (row %) |  |  |  | 1.000 |  |  |  |  | 0.745 |
| Typical only | 2 (22.2) | 4 (44.4) | 3 (33.3) |  |  | 0 | 0 | 1 (100) |  |
| Atypical only | 1 (12.5) | 4 (50.0) | 3 (37.5) |  |  | 3 (9.7) | 17 (54.8) | 11 (35.5) |  |
| Both | 0 | 3 (75) | 1 (25) |  |  | 2 (40.0) | 1 (20.0) | 2 (40.0) |  |
| None | 2 (9.1) | 11 (50.0) | 9 (40.9) |  |  | 0 | 4 (66.7) | 2 (33.3) |  |
| Daily CPZE dose^4^, mean ± SD | 282.3 ± 202.1 | 284.1 ± 109.7 | 239.3 ± 81.5 | 0.403 |  | 566.7 ± 372.1 | 344.4 ± 162.6 | 362.3 ± 202.6 | 0.863 |
| NL: yes, n (row %) | 3 (14.3) | 11 (52.3) | 7 (33.3) | 0.835 |  | 5 (13.5) | 18 (48.7) | 14 (37.8) | 0.848 |
|  |  |  |  |  |  |  |  |  |  |
| **Mood Stabilizing Medications** |  |  |  |  |  |  |  |  |  |
| Mood stabilizers, yes, n (row %) | 0 | 3 (100) | 0 | 0.344 |  | 5(11.9) | 21 (50.0) | 16(38.1) | 1.000 |
| VPA: yes, n (row %) | 0 | 1 (100) | 0 | 1.000 |  | 2 (9.5) | 11 (52.4) | 8 (38.1) | 1.000 |
|  |  |  |  |  |  |  |  |  |  |
| **Other Medications** |  |  |  |  |  |  |  |  |  |
| Benzodiazapines: yes, n (row %) | 3 (15.8) | 10 (52.6) | 6 (31.6) | 0.542 |  | 4 (22.2) | 4 (22.2) | 10 (55.6) | 0.055 |
| Antidepressants: yes, n (row %) | 0 | 2 (66.7) | 1 (33.3) | 1.000 |  | 1 (5.6) | 7 (38.9) | 10 (55.6) | 0.055 |
| ^1^ p-value when comparing M allele carriers to ValVal patients.  ^2^ Negative Symptoms (BPRS items 3 + 13 + 14 +16+ 18)  ^3^ Positive Symptoms (BPRS items 4 + 7 + 8 +10 + 11 + 12 +15 + 17)  ^4^ CPZE: Chlorpromazine equivalent dose. | | | | | | | | | |

Supplementary Figure 1. Fasting Plasma Proline is significantly associated with Schizophrenia but not Bipolar Disorder.

We previously reported that patients with schizophrenia (n=64) had significantly higher fasting plasma proline as compared to a sample of matched control subjects (n=90) (Clelland et al., 2011). An association between plasma proline and both bipolar disorder and schizoaffective disorder has also been reported, however the finding in bipolar patients may have been confounded by current valproate (VPA) treatment (Jacquet et al., 2005). Therefore, we tested for an associated between fasting plasma proline (>8 hours of fasting) and bipolar disorder in a new sample of 40 bipolar disorder patients, who were not receiving VPA. Subjects were recruited upon presentation at the Bellevue Hospital Center Comprehensive Emergency Psychiatric Program (CPEP), and were included if they had an admission diagnosis of bipolar disorder, and had not recently received treatment with VPA, as confirmed via blood level testing. A subset of these bipolar patients (n=13) qualified for inclusion in the full study detailed in the manuscript, as they also had a follow-up study visit.

Levels of fasting plasma proline (umol/L) is shown for each group in Figure S1 (following page). Patients with bipolar disorder did not have proline levels significantly different to control subjects (Mann-Whitney z = 0.111, p = 0.9116). To adjust for previously reported proline gender differences we also assessed hyperprolinemic status, defined according to Jacquet et al., as a proline level two standard deviations (SDs) or more above the gender-specific mean of controls (Jacquet et al., 2005). While hyperprolinemia was present in 26.6% of schizophrenia patients (17/64), the proportion of bipolar disorder patients exhibiting peripheral hyperprolinemia (3/40; 7.5%) was not significantly different to controls (5/90; 5.6%) Fisher’s exact p=0.70. This data supports the body of work demonstrating a positive, but specific association between hyperprolinemia and schizophrenia-spectrum disorders (Jacquet et al., 2005; Tomiya et al., 2007, Clelland et al., 2011; Oresic et al., 2011).

**
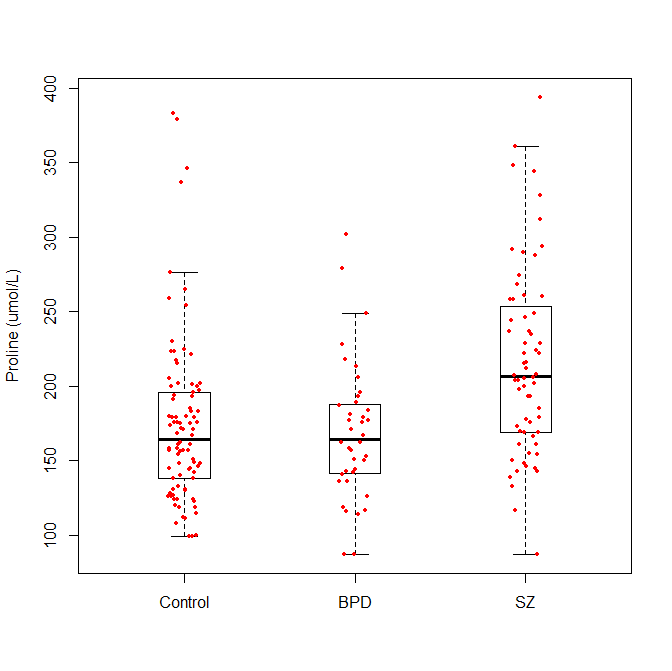
**

**Figure 1. Proline in Psychiatric Illness.** Fasting plasma proline was plotted for control subjects (mean±SD: 174.28±55.97, n=90), bipolar disorder (BPD) patients (mean±SD: 168.75 ±45.50, n=40) and schizophrenia (SZ) patients (mean±SD: 215.84±63.00, n=64). Red jittered points represent individual data. The horizontal line within each box represents the group median. The box indicates the IQR. The whiskers extend to the most extreme data point which is 1.5x the IQR.

| **Supplementary Table 2: Multivariate Analysis of Schizophrenia Symptom Outcomes (n=95).** | | | | | |  | | | |
| --- | --- | --- | --- | --- | --- | --- | --- | --- | --- |
| ***Dependent* and Independent Variables** | **Test Value** | **F-test** | **p** | **β (95% CI)** | **p** |  | | | |
| ***BPRS, SAPS and SANS Score*** |  |  |  |  |  |  | | | |
| Interaction (COMT x proline) | WL 0.69 | F_(2,174)_=6.0 | <0.0001 |  |  |  | | | |
| *BPRS* |  | F_(2,89)_=0.42 | 1.00^a^ |  |  |  | | | |
| *SAPS* |  | F_(2,89)_=2.97 | 0.168^a^ |  |  |  | | | |
| *SANS* |  | F_(2,89)_=13.33 | <0.001^a^ |  |  |  | | | |
|  |  |  |  |  |  |  | | | |
| ***BPRS^b^ R^2^=0.018, p=1.0^a^*** |  |  |  |  |  |  | | | |
| Interaction |  |  |  |  |  |  | | | |
| Val/Val x proline *versus* Val/Met x proline |  |  |  | -0.02 (-0.07,0.02) | 0.362^c^ |  | | | |
| Val/Val x proline *versus* Met/Met x proline |  |  |  | -0.01 (-0.06,0.39) | 0.712^c^ |  | | | |
|  |  |  |  |  |  |  | | | |
| ***SAPS^b^ R^2^=0.150, p=0.035^a^*** |  |  |  |  |  |  | | | |
| Interaction |  |  |  |  |  |  | | | |
| Val/Val x proline *versus* Val/Met x proline |  |  |  | -0.07 (-0.13,0.01) | 0.023^c^ |  | | | |
| Val/Val x proline *versus* Met/Met x proline |  |  |  | -0.04 (-0.98,0.14) | 0.144^c^ |  | | | |
|  |  |  |  |  |  |  | | | |
| ***SANS^b^ R^2^=0.259, p=0.0003^a^*** |  |  |  |  |  |  | | | |
| Interaction |  |  |  |  |  |  | | | |
| Val/Val x proline *versus* Val/Met x proline |  |  |  | 0.12 (0.04,0.20) | 0.004^c^ |  | | | |
| Val/Val x proline *versus* Met/Met x proline |  |  |  | 0.19 (0.11,0.26) | <0.001^c^ |  | | | |
| ^a^ Bonferroni adjusted p-values.  ^b^ Multivariate model included three dependent variables, plus main and interaction effects.  ^c^ Unadjusted p-values.  WL: Wilk’s Lambda | | | | | |  |  |  |  |

Supplementary Figure 2. The Relationship between Proline and *COMT* Genotype on Negative Symptoms in Schizophrenia.

The scatterplots below show the relationship between proline and total negative symptoms, as assessed using the Scale for Negative Symptoms (SANS), by *COMT* genotype (Met Allele or Val/Val). There is a significant positive relationship between total SANS score and proline in schizophrenia patients with the Met allele (Spearman’s rho= 0.36, p=0.009, n=53), while conversely there is a significant negative relationship between total SANS score and proline in Val/Val schizophrenia patients (Spearman’s rho= -0.47, p=0.0019, n=42).

**Figure S2 The Relationship between Proline and *COMT* Genotype on Negative Symptoms in Schizophrenia.**

| **Supplementary Table 3: Prediction of Negative Symptoms from Proline Level and *COMT* in African-American, Caucasian, and Hispanic Schizophrenia Patients.** | | | | | | |
| --- | --- | --- | --- | --- | --- | --- |
| ***Independent* and Dependent Variables** | **β Coefficient** | **SE** | | **T** | **p-value** |  |
| ***Total SANS Score*** | | | | | | |
| **African American^a^(n=37)** |  | |  |  |  |  |
| Proline | -0.1207 | | 0.0495 | -2.44 | 0.002* |  |
| COMT (ordinal^b^ Val/Val, Val/Met, Met/Met) | -14.1884 | | 7.3528 | -1.93 | 0.062 |  |
| Interaction (Proline x COMT) | 0.0651 | | 0.0295 | 2.21 | 0.034* |  |
|  |  | |  |  |  |  |
| **Caucasian^a^ (n=28)** |  | |  |  |  |  |
| Proline | -0.1807 | | 0.0840 | -2.15 | 0.042* |  |
| COMT (ordinal^b^ Val/Val, Val/Met, Met/Met) | -20.8422 | | 9.6946 | -2.15 | 0.042* |  |
| Interaction (Proline x COMT) | 0.1239 | | 0.0398 | 3.12 | 0.005* |  |
|  |  | |  |  |  |  |
| **Hispanic^a^ (n=30)** |  | |  |  |  |  |
| Proline | -0.1845 | | 0.0632 | -2.92 | 0.007* |  |
| COMT (ordinal^b^ Val/Val, Val/Met, Met/Met) | -23.9559 | | 7.6983 | -3.11 | 0.004* |  |
| Interaction (Proline x COMT) | 0.1009 | | 0.0303 | 3.33 | 0.003* |  |
|  |  | |  |  |  |  |
| **^a^** Self-reported.  ^b^ Ordinal genotype due to the relationship documented in manuscript Fig 1a. | | | | | | |

|  | **Supplementary Table 4: Prediction of Negative Symptoms from Proline Level and COMT, with Stratification and Adjustment for Illness Duration.** | | | | | |  | |
| --- | --- | --- | --- | --- | --- | --- | --- | --- |
| ***Independent* and Dependent Variables** | | **β Coefficient** | **SE** | | **T** | **p-value** | |  |
| ***Total SANS Score*** | | | | | | | | |
| **Stratified Models^a^** | |  | |  |  |  | |  |
| **Short Illness Duration (n=30)** | |  | |  |  |  | |  |
| Proline | | 0.1589 | | 0.0515 | 3.09 | 0.005* | |  |
| COMT (Met Allele versus Val/Val) | | 43.9870 | | 17.898 | 2.46 | 0.021* | |  |
| Interaction (Proline x COMT) | | -0.2381 | | 0.0757 | -3.14 | 0.004* | |  |
|  | |  | |  |  |  | |  |
| **Long Illness Duration (n=30)** | |  | |  |  |  | |  |
| Proline | | 0.0781 | | 0.0404 | 1.93 | 0.064 | |  |
| COMT (Met Allele versus Val/Val) | | 52.416 | | 12.059 | 4.35 | <0.001* | |  |
| Interaction (Proline x COMT) | | -0.1916 | | 0.0488 | -3.93 | 0.001* | |  |
|  | |  | |  |  |  | |  |
| **Adjusted Model (n=60)** | |  | |  |  |  | |  |
| Proline | | 0.1290 | | 0.0345 | 3.73 | <0.001* | |  |
| COMT (Met Allele versus Val/Val) | | 47.266 | | 10.71 | 4.41 | <0.001* | |  |
| Interaction (Proline x COMT) | | -0.2151 | | 0.0437 | -4.92 | <0.001* | |  |
| Duration of Illness | | 0.1798 | | 0.1503 | 1.20 | 0.237 | |  |
|  | |  | |  |  |  | |  |
| **^a^** The total sample with a measure of illness duration (n=60) was bisected based upon the median illness duration (12.5 years). Those with duration <12.5 years were considered short illness duration (n=30, median=4 years), and those > 12.5 years considered long illness duration (n=30, median=23.5 years). | | | | | | | | |

| **Supplementary Table 5. Bivariate Association Between Schizophrenia Patient Demographic and Clinical Characteristics, with SANS Total Score, n=95** | | | | |
| --- | --- | --- | --- | --- |
| **Characteristic** | | β (95% CI) |  | p-value^a^ |
| Gender^b^ | -1.697 (-7.609, 4.215) | |  | 0.570 |
| Ethnicity^c^ |  | |  |  |
| African American v Caucasian | 6.059 (-1.026, 13.143) | |  | 0.093* |
| African American v Hispanic | 7.028 (0.079, 13.977) | |  | 0.047* |
| Age | 0.024 (-0.239, 0.287) | |  | 0.857 |
| Alcohol Dependence/abuse^c^ |  | |  |  |
| None v Abuse | -0.236 (-9.775, 9.303) | |  | 0.961 |
| None v Dependence | -9.505 (-18.023, -0.987) | |  | 0.029* |
| Duration of Illness^d^ | 0.102 (-0.239, 0.443) | |  | 0.552 |
| Hospital Duration^e^ | 0.026 (-0.121, 0.172) | |  | 0.729 |
| Daily CPZE dose^f^ | 0.004 (-0.005, 0.014) | |  | 0.353 |
| Neuroleptic (NL) Type^c,g^ |  | |  |  |
| Atypical v Typical | 2.082 (-5.763, 9.930) | |  | 0.599 |
| Atypical v both | -1.584 (-9.430, 6.261) | |  | 0.689 |
| VPA Treatment^h^ | -0.578 (-7.157, 6.001) | |  | 0.862 |
| Benzodiazapines | -3.342 (-10.713, 4.028) | |  | 0.370 |
| **^a^ * =** Taken forward into multivariate model.  ^b^ Binary variable: Male v female.  **^c^** For categorical analysis the reference category is the first level listed for each variable.  ^d^Years since first hospitalization and blood draw, n=60 for whom this characteristic could be obtained.  **^e^**Days in hospital prior to fasting blood draw and symptoms assessment.  **^f^** CPZE: Chlorpromazine equivalent dose, n=93 (as one subject’s NL had no CPZ equivalent, and one subjects did not receive a NL).  **^g^** n=94 (as one subject did not receive a NL).  **^h^** Binary variable: no versus yes, n=92 (as three subjects had received <48 hours of VPA treatment). | | | | |

| **Supplementary Table 6. Bivariate Association Between Bipolar Disorder Patient Demographic and Clinical Characteristics, with Percent Change in Negative Symptoms, n=43** | | |
| --- | --- | --- |
| Characteristic (at Visit 2) | β (95% CI) | p-value^a^ |
| Gender^b^ | 1.359 (0.003, 0.268) | 0.045* |
| Ethnicity^c^ |  |  |
| African American v Caucasian^d^ | -0.048 (-0.022, 0.121) | 0.566 |
| African American v Hispanic | -0.272 (-0.473, -0.071) | 0.009* |
| Age | 0.003 (-0.004, 0.010) | 0.369 |
| Duration of Illness^e^ | -0.001 (-0.011, 0.009) | 0.828 |
| Alcohol Dependence/abuse^c^ |  |  |
| None v Abuse | -0.020 (-0.174, 0.133) | 0.790 |
| None v Dependence | -0.055 (-0.240, 0.130) | 0.554 |
| Duration (days) between symptom assessments | 0.013 (-0.002, 0.028) | 0.082* |
| Daily CPZE dose^f^ | -0.000 (-0.000, 0.000) | 0.607 |
| Neuroleptic (NL) Type^c^ |  |  |
| None v Atypical | -0.091 (-0.285, 0.103) | 0.348 |
| None v Typical | -0.006 (-0.476, 0.465) | 0.981 |
| None v both | -0.230 (-0.494, 0.033) | 0.085* |
| VPA Treatment | -0.013 (-0.148, 0.122) | 0.845 |
| Benzodiazapines | -0.120 (-0.251, 0.011) | 0.072* |
| Antidepressants | 0.004 (-0.133, 0.140) | 0.958 |
| **^a *^**Taken forward into multivariate model.  **^b^** Binary variable: Male v female.  **^c^** For categorical analysis the reference category is the first level listed for each variable.  **^d^** Includes n=1 Asian subject. Parameter estimates did not change following the removal of this subject, and so this subject was included in all final models, within the largest ethnicity group.  ^e^ Years since first hospitalization and blood draw, n=35 for whom this characteristic could be obtained.  ^f^ CPZE: Chlorpromazine equivalent dose, n=37 (as six subjects did not receive a NL). | | |

**Supplementary Figure 3. Negative Symptom Change in Bipolar Disorder Patients Treated with VPA, by *COMT* Genotype.**

VPA-treated bipolar disorder patients had significantly higher fasting plasma proline (mean: 268.29±49, n=21) than those who did not receive VPA (mean 176.22±101, n=22) (z=-3.60, p=0.0003). Additionally, Val/Val VPA-treated bipolar disorder patients had a greater overall percent reduction in negative symptoms (mean: -15.6±10, n=8) as compared to Met allele carriers (mean: -5.6±28, n=13), as shown in Figure S3. However, this result did not reach statistical significance (Mann-Whitney z = 0.95, p = 0.34), likely due to the small sample (n=13 versus n=8).


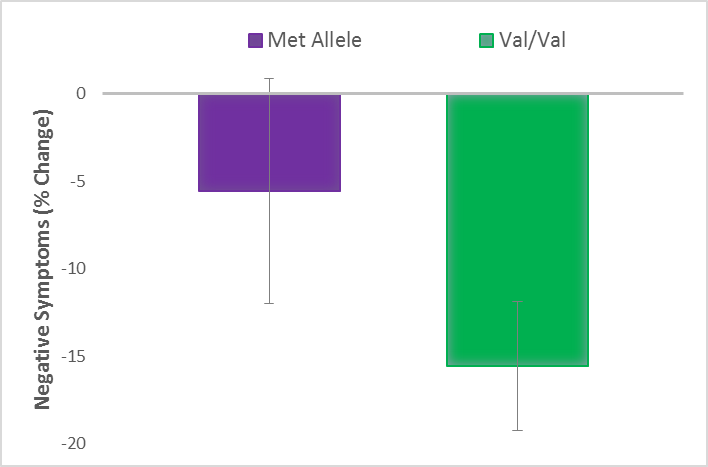


**Figure S3 The Relationship between Percent Change in Negative Symptoms and *COMT* Genotype in VPA-treated Bipolar Disorder Patients.**  The bar chart shows the mean (±SEM) percent change in symptoms for Met allele carriers and Val/Val bipolar patients. The percent change in symptoms was calculated as: {total negative symptoms subscale at visit 2 – total negative subscale at visit 1}/total negative subscale at visit 1 x 100%. SEM= standard error of the mean.

**Supplementary References.**

Clelland CL, Read LL, Baraldi AN, Bart CP, Pappas CA, Panek LJ, Nadrich RH, Clelland JD: Evidence for association of hyperprolinemia with schizophrenia and a measure of clinical outcome. Schizophr Res 2011; 131(1-3):139-45

Jacquet H, Demily C, Houy E, Hecketsweiler B, Bou J, Raux G, Lerond J, Allio G, Haouzir S, Tillaux A, Bellegou C, Fouldrin G, Delamillieure P, Ménard JF, Dollfus S, D'Amato T, Petit M, Thibaut F, Frébourg T, Campion D: Hyperprolinemia is a risk factor for schizoaffective disorder. Mol Psychiatry 2005; 10(5):479-85

Orešič M, Tang J, Seppänen-Laakso T, Mattila I, Saarni SE, Saarni SI, Lönnqvist J, Sysi-Aho M, Hyötyläinen T, Perälä J, Suvisaari J: Metabolome in schizophrenia and other psychotic disorders: a general population-based study. Genome Med 2011; 3(3):19

Tomiya M, Fukushima T, Watanabe H, Fukami G, Fujisaki M, Iyo M, Hashimoto K, Mitsuhashi S, Toyo'oka T: Alterations in serum amino acid concentrations in male and female schizophrenic patients. Clin Chim Acta 2007; 380(1-2):186-90
